# Molecular Characterization and Functional Analysis of *Pomacea canaliculata* Mos Gene (*PcMos*): A Molecular Switch of Female Fecundity

**DOI:** 10.3390/biology15191774

**Published:** 2026-10-06

**Authors:** Haotian Gu, Haiyuan Teng, Tianshu Zhang, Yongda Yuan

**Affiliations:** 1 Shanghai Key Laboratory of Protected Horticultural Technology, Eco-Environmental Protection Research Institute, Shanghai Academy of Agricultural Sciences, Shanghai 201403, China; guhaotian@saas.sh.cn (H.G.);; 2 Shanghai Engineering Research Centre of Low-Carbon Agriculture (SERCLA), Shanghai 201415, China

**Keywords:** PcMos, RNAi, endocrine hormone, MAPK signaling, vitellogenin, fecundity

## Abstract

The invasive *Pomacea canaliculata* has emerged as a notorious pest that endangers aquatic crops, ecological services and functioning. Although well-documented as a mammalian oocyte maturation factor, the biological roles of Moloney sarcoma oncogene (*Mos*) in invertebrates, notably *P. canaliculata*, remain unfathomed. We integrated RNA interference (RNAi)-mediated gene silencing alongside bioinformatic, molecular, biochemical and histological approaches to dissect fecundity-related functions and modulatory mechanisms of PcMos at the mRNA, protein and in silico levels. Characterized by the conserved protein kinase domain and motif, the PcMos protein sequence presented close phylogeny with its molluscan counterparts. Spatiotemporal expression profiling showed that mRNA and protein levels of PcMos were enriched in female adults and ovarian tissues. Efficient in vivo RNAi effects were induced as the transcription and translation of PcMos significantly declined by 78.2% and 79.0%, respectively, following a microinjection of 5 μg dsPcMos per female post 48 h. The downstream MAPK pathway, comprising MEK1, ERK1 and p90RSK1, was depressed when subjected to dsPcMos treatment, as reflected by pronounced decreases in mRNA and phosphorylated protein levels. Substantial diminution of E2/Pg/LH/FSH contents were noticed in the *PcMos*-RNAi group versus the dsEGFP group, suggesting fundamental implications of *PcMos* in endocrine regulation. In addition, the concurrent expression and high affinity between PcMos and VTG were visualized by the immunofluorescence and molecular docking, which may be associated with the diminished VTG content and expression upon *PcMos* silencing. Moreover, *PcMos* knockdown led to impaired ovarian development and reduced SP content, the number ofmature oocytes, egg production and egg-hatching rates. Taken together, our findings pointed to *PcMos* as a molecular switch pivotal for female fecundity, potentially acting through coordinated effects on MAPK signaling, endocrine hormone homeostasis and yolk deposition. This study may inspire the precision pest management and inform the design of RNAi-based sterility agents to suppress *P. canaliculata* populations.

## Simple Summary

Mos gene is pivotal for oocyte meiosis and maturation in mammals. The highly fecund *Pomacea canaliculata* has rapidly invaded and infested non-native regions, jeopardizing paddy fields and agroecosystems. Herein, we investigated the biological functions of *PcMos*. PcMos was evolutionarily conserved among molluscan orthologs and displayed female-biased expression profiles. *PcMos*-RNAi disrupted ovarian endocrine hormones and yolk accumulation, with diminished levels of E2/Pg/LH/FSH alongside VTG expression and SP content. *PcMos* silencing also arrested oocyte maturation, impaired ovarian development, and reduced egg-laying and hatchability. Moreover, it attenuated the phosphorylated activation of MAPK signaling. This work yielded insights into *PcMos* modulation in female reproductive aspects and highlighted it as a promising molecular target for RNAi-based female sterilant.

## 1. Introduction

*Pomacea canaliculata* (Gastropoda: Ampullariidae) (Lamarck, 1822), also termed the golden apple snail (GAS), is a cosmopolitan invasive freshwater gastropod native to South America [1]. Featuring polyphagous feeding habits, voracious appetite, rapid growth, robust fecundity and strong adaptability, this mollusc has thrived as an agricultural pest that causes devastating damage to paddy fields, wetland biodiversity and ecosystems [2,3]. Since *P. canaliculata* infestations incur massive ecological consequences and economic losses, it has been listed among China’s first cohort of invasive alien species (IAS) and the world’s 100 worst IAS [4,5]. Its unique reproductive traits, notably dioecious mating, long-term sperm storage and an r-selected life history, are physiological cornerstones responsible for the aggressive expansion and successful establishment of *P. canaliculata* populations in non-indigenous ranges [6,7]. After lengthy copulation, females can store viable sperm in seminal receptacles for several months and continuously lay multiple clutches of fertilized eggs into a novel habitat, independently triggering a severe invasion [6]. In addition, their frequent promiscuous mating significantly enhances the genetic diversity of offspring, thereby facilitating rapid adaptation to changing environments [8]. These reproductive advantages are further reinforced by the *r*-selected life-history strategy. *r* strategists are typically characterized by short life cycles and high reproductive output, which allow rapid population growth under favorable conditions [9]. The invasion pattern of *P. canaliculata* closely reflects these *r*-strategy traits, granting its strong environmental tolerance and considerable phenotypic plasticity [10]. It has been estimated that GAS females can produce 2–3 generations per annum and lay circa 13,764 eggs during their lifespan [11].

Thus far, various biological, physical and chemical approaches have been proposed for GAS management. Among these, chemical molluscicides such as niclosamide and metaldehyde, are widely adopted and stand out as the mainstay practice due to their high efficacy [12,13]. However, their indiscriminate and excessive use may exert detrimental effects on nontarget aquatic biota and ecosystems [14,15]. This scenario is also true for the novel potent molluscicide, tea saponin, which can trigger moderate toxicity to zebrafish and pronounced hepatotoxicity plus systemic dysregulation in Hybrid grouper albeit its botanical nature [16,17]. To address adverse environmental consequences caused by the existing molluscicides, ecofriendly, precise and sustainable alternatives are desperately encouraged.

Extensively used as a reverse genetics tool, RNA interference (RNAi) has witnessed burgeoning applications in gene function studies and agricultural pest control over recent decades [18]. With advantages such as high specificity and efficiency, low ecological risk and precise mode of actions, RNAi-based biopesticides are acclaimed by Science (2024) [19] as one of the top ten scientific breakthroughs, ushering in the third revolution for pesticide development [20]. Mounting investigations have established the robust and persistent RNAi response for *P. canaliculata*. The specific and effective RNAi response is induced by either double-stranded RNA (dsRNA) or small interfering RNA (siRNA) that are delivered via microinjection, ingestion or immersion [1,3,5,21,22]. To further enhance the efficacy and stability in aquatic environments, Liu et al. [5] formulated @PEG-PLys (SH) nanoparticle complexes as the carrier to encapsulate naked dsRNA, enabling practical implementation of biopesticides derived from RNAi.

In oviparous animals, the accumulation of yolk proteins (vitellogenesis) and the production of mature eggs (oogenesis) are essential reproductive processes [23]. The regulation of vitellogenesis, oogenesis and reproductive fitness is sophisticatedly orchestrated by a range of endogenous elements like hormonal and nutritional factors alongside a molecular basis [24,25]. Maintenance of hormonal homeostasis is crucial for normal physiological activities and biological processes. Sex hormone synthesis and secretion during the pubertal period affect reproductive organogenesis, sexual maturation and fecundity in *P. canaliculata* [26,27]. In addition, ovarian nutrition is recognized as the determinant for oocyte maturation and reproductive investment, with better-nourished ovaries allocating more internal resources (primarily yolk proteins) to drive successful oocyte growth, egg deposition and embryonic development [28,29]. Mechanistically, the classical mitogen-activated protein kinase (MAPK) signaling cascade is evolutionarily conserved and responsible for gametogenesis, oocyte maturation and reproduction in vertebrates [30,31]. Specifically in the nematode (*Caenorhabditis elegans*), it governs oogenesis progression and is a hallmark of oocyte aging [32]. In addition, gonad-specific expressions of MAPK cascade genes are noted in the noble scallop (*Chlamys nobilis*), highlighting potential roles of the MAPK pathway in oogenesis, gonad development and reproduction [33]. Collectively, unraveling the coordinated regulation of hormonal signals and yolk deposition dynamics, along with the molecular mechanisms underpinning female fertility can contribute to the conception of effective countermeasures against *P. canaliculata*.

Screening desirable candidate genes involved in survival, growth, development and pivotal biological processes as silencing targets is the prerequisite for effective RNAi elicitation [34]. The Moloney sarcoma oncogene (*Mos*) encodes a serine/threonine kinase that activates the MAP kinase (MAPK) cascade through direct phosphorylation of MEK [35]. Identified as a cell cycle regulator and oocyte maturation factor [36,37], *Mos* is abundantly transcribed in vertebrate oocytes and its mutation induces early embryonic arrest and fragmentation alongside female infertility disorders by blocking the ERK pathway [38,39]. Despite its potential implications in gonadal development of a bivalve mollusc [36], the biological role and regulatory network of the Mos gene in the gastropod *P. canaliculata* (*PcMos*), notably in terms of female reproductive fitness, constitute a significant knowledge gap.

Building upon insights gained from the previous literature, we hypothesized that *PcMos* also modulated female reproductive physiology and its depletion might render the sterile phenotype in female *P. canaliculata*. qRT-PCR, Western blotting, ELISA, immunofluorescence, H&E staining and fecundity assays coupled with RNAi were utilized to verify that assumption. The objectives of this study were: (1) to confirm the fundamental roles of *PcMos* in female fecundity; (2) to decipher how *PcMos* deficiency affects ovarian MAPK signaling, endocrine homeostasis and yolk reserves.

## 2. Materials and Methods

### 2.1. Snail Husbandry and Collection

Adult individuals (7.72 ± 0.64 g) with uniform body sizes were originally handpicked from a pond in Zhuanghang Town, Shanghai City, China (coordinates 30°53′29″ N, 121°23′16″ E). They were identified as *P. canaliculata* by morphological characterization and DNA barcoding of cytochrome c oxidase subunit I (COI) genes [40]. During the 7-day acclimation period, snails were kept in aquariums (80 cm long × 60 cm wide × 60 cm tall) containing dechlorinated water, reared with fresh lettuce daily under conditions of 24 ± 2 °C, 12 h light:12 h dark photoperiod and natural illumination. We adopted the method of Gamarra-Luques et al. [41] to distinguish sexes, with individuals harboring a concave operculum classified as females. Snails at different developmental stages were the progeny from the same batch of adults. A fraction of egg clutches laid by female adults were stripped off from the walls of tanks and harvested, while most others were left to hatch, fed following the above regime and sacrificed after they grew into hatchlings (0.3 ± 0.1 g) and juveniles (1.5 ± 0.3 g). The tanks were covered with nylon nets, and tap water was purified using an overflow filter that was replenished weekly. Dead snails and food residue were cleaned daily.

### 2.2. Sequence Alignments and Phylogenetic Analysis

Amino acid and nucleotide sequences of PcMos were retrieved from the NCBI GenBank databases (https://www.ncbi.nlm.nih.gov/, accessed on 20 April 2026). Mos homologs from molluscs were searched by the blastp program using >XP_025095407.1 as a query (https://blast.ncbi.nlm.nih.gov/Blast.cgi, accessed on 20 April 2026). Multiple sequence alignments were performed by GeneDoc v2.7.0 (http://nrbsc.org/gfx/genedoc/index.html, accessed on 21 April 2026) plus ClustalX v1.84 (https://clustal-x.software.informer.com/, accessed on 21 April 2026). The cladogram of PcMos and its orthologues was constructed using the neighbor-joining (NJ) method in MEGA 7.0 with 1000 bootstrap replicates (http://megasoftware.net/, accessed on 21 April 2026), and we used ChiPlot to beautify the phylogenetic tree (https://www.chiplot.online/, accessed on 21 April 2026).

### 2.3. Total RNA Extraction and dsRNA Synthesis

Specimens were homogenized using an SKXL homogenizer (BiHeng Biotechnology, Shanghai, China) at 4 °C, followed by total RNA isolation with the SV Total Isolation System Kit plus genomic DNA (gDNA) Eraser (Promega, Madison, WI, USA). RNA quantity and quality were assessed using a Nanodrop 2000 spectrophotometer (Thermo Fisher Scientific, Waltham, MA, USA). RNA integrity was examined by 1% formaldehyde agarose gel electrophoresis.

Primers for dsRNA synthesis were designed using the Primer-BLAST tool (http://www.ncbi.nlm.nih.gov/tools/primer-blast/, accessed on 10 March 2026, Table S1) and synthesized by Sangon Biological Engineering Technology Company (Shanghai, China). A 354-bp fragment of *PcMos* was amplified using primers that contained T7 RNA polymerase promoter sequences at the 5′ ends (Table S1). The synthesis and purification of dsPcMos were performed according to the manufacturer’s protocol of the T7 RiboMAX^TM^ Express RNAi System Kit (Promega, Madison, WI, USA). As the negative control, lyophilized dsEGFP (5 mg, 431 bp) was procured from Shanghai Plant Science Biotechnology Co., Ltd. (Shanghai, China). Final concentrations of both dsEGFP and dsPcMos were determined via a NanoDrop 2000 spectrophotometer (Thermo Fisher Scientific, Waltham, MA, USA) and diluted to a working concentration of 1 µg/µL.

### 2.4. cDNA Synthesis and qRT-PCR Analysis

Total RNA was isolated as outlined in Section 2.3. First-strand cDNA synthesis was performed using the PrimeScript^TM^ RT Reagent Kit with gDNA Eraser (Takara, Tokyo, Japan). Briefly, 1.0 µg RNA was mixed with 4 µL 5 × PrimeScript RT Master Mix and adjusted to 20 µL with nuclease-free ddH_2_O. Reverse transcription was carried out in a 9902 Applied Biosystems thermal cycler (Life Technologies, Foster, CA, USA) under the following conditions: 42 °C for 15 min, followed by 85 °C for 5 s. The yielded cDNA was diluted to 100 µL with ddH_2_O and stored at −20 °C until use.

qRT-PCR reactions were assembled in 20 µL volumes using the SYBR Color qPCR Master Mix (Vazyme, Nanjing, China), consisting of 10 µL SYBR Green mix, 0.5 µL each of forward and reverse primers (10 µM), 2 µL diluted cDNA template, and 7 µL ddH_2_O. Amplification was run on a CFX96^TM^ Real-Time PCR Detection System (Bio-Rad, Hercules, CA, USA) with the following thermal profile: initial denaturation at 95 °C for 3 min, followed by 40 cycles of 95 °C for 10 s and 60 °C for 30 s. A melt curve analysis was conducted (60–95 °C, ramping at 0.5 °C per 5 s) to verify amplicon specificity and exclude primer dimers or genomic DNA contamination. Gene-specific primers were designed using the Primer-BLAST tool (http://www.ncbi.nlm.nih.gov/tools/primer-blast/, accessed on 10 March 2026) and were detailed in Table S1. GAPDH was selected as the reference gene based on previous studies validating its high expression stability across various reproductive states in *P. canaliculata* [42]. The relative expression of each gene was normalized using GAPDH and threshold cycle (Ct) values, according to the 2^−∆∆CT^ method developed by Livak and Schmittgen [43]. Two technical replications were set for each sample, with three independent biological samples for each assay.

### 2.5. Sample Collection and RNAi Assay

#### 2.5.1. Specimens for Expression Profiling

Samples were collected for qRT-PCR (Section 2.4) and blotting analysis (Section 2.6) across different developmental stages and tissues. For developmental stages, eggs, hatchlings, juveniles, females and males were sacrificed, with two individuals or one egg mass per replicate and three replicates per sample. For tissue analysis, the foot, intestine, digestive gland, gill, mantle, albumen gland and ovary of female adults were dissected and pooled, with three samples per replicate and three replicates per sample.

Although the ovary is attached to the surface of the digestive gland, it can be visually distinguished by the milky white or whitish color from the yellowish-brown and reticulated digestive gland [41,44]. To prevent tissue cross-contamination, all dissections were performed meticulously under a stereoscopic microscope (Olympus SZX17, Olympus Corporation, Tokyo, Japan), utilizing these color and textural differences to carefully peel the ovarian tissue away from the digestive gland. Moreover, the quality control for ovarian tissue purity was conducted and detailed in Text S1.

#### 2.5.2. dsRNA Delivery Protocol

The dsRNA injection proceeded as per our previous descriptions [1]. Prior to injection, snails were anesthetized by placing them on ice for 15–20 min. Afterwards, the operculum was carefully retracted, and dsRNA solution (1 µg/µL) was administered into the foot muscle (5 µg/snail) using a sterile Hamilton 701 RN microsyringe (Hamilton, Whittier, CA, USA). The needle was maintained in position for 10 s post-injection to avoid solution leakage. A control injection was implemented with an equivalent volume of dsEGFP comparable to the dsPcMos treatment.

#### 2.5.3. Sampling of RNAi-Females for Functional Characterization

Adult females received a single injection of dsRNA (5 µg per snail) and were recruited 48 h later, with at least 50 individuals (*n* = 50) for each group (*PcMos*-RNAi and dsEGFP control). Specifically, for biochemical tests, three ovaries were harvested as one sample, with three samples per group (*n* = 3). For the fecundity bioassay, ten mating parallels were assigned to each group (*n* = 10), with each parallel composed of one male and one female. For the hematoxylin and eosin staining, three ovaries were dissected from each treatment (*n* = 3), and each tissue represented one observation. For qRT-PCR analysis of relative gene expression, three ovaries were pooled as one sample, and each treatment consisted of three samples (*n* = 3). For Western blotting and immunofluorescence assays, each assay comprised six ovaries, three for each group (*n* = 3), with one ovary corresponding to one measurement.

### 2.6. Protein Extraction and Western Blotting

Total protein isolation and blotting were conducted as previously described [3]. Circa 100 mg tissues were homogenized and lysed in 400 µL RIPA lysis buffer (CWBio, Beijing, China) with 1×EDTA-Free Protease Inhibitor Cocktail (Bimake, Houston, TX, USA) and 1× Phosphatase inhibitor cocktail A (Beyotime, Shanghai, China), incubated on ice for 30 min and centrifuged for 15 min at maximum speed to remove the insoluble fraction. Protein abundance was quantified as per the bicinchoninic acid (BCA) method (CWBIO, Beijing, China) with equal amounts of protein loaded and separated by 4% stacking gel plus 10% resolving gel in a Mini-Protean apparatus (Bio-Rad, Hercules, CA, USA) at 120 V for 100 min. Gels were transferred to nitrocellulose membranes (0.45 µm, Beyotime, Shanghai, China) post SDS-PAGE, rinsed with Tris-buffered saline (TBS) for 5 min. Blots were blocked at room temperature for 1 h in TBS containing 0.1% Tween 20 (TBST) and 5% (*w*/*v*) skim milk, followed by washing 3 × 5 min with TBST. Blots were incubated overnight at 4 °C with the primary antibody diluted in TBST, washed 3 × 10 min in TBST and probed with HRP-Conjugated Goat Anti-Rabbit IgG (Beyotime, Shanghai, China) as the secondary antibody in TBST (1:3000). Upon extensive rinses, the immunoreactivity was detected by an enhanced chemoluminescence (ECL) kit (Beyotime, Shanghai, China) in a Molecular Imager ChemiDoc XRS System (Bio-Rad, Hercules, CA, USA). Gray values of protein bands were quantified by densitometry in ImageJ software (version 1.53, Wayne Rasband, National Institutes of Health, MD, USA) and normalized to the loading control (GAPDH). The primary antibodies and diluted concentrations were listed in Table S2. Antibody specificity was validated by the detection of dominant bands at the predicted molecular weights. Routine negative controls were performed by omitting primary antibodies, which yielded no background signals.

### 2.7. Biochemical Measurements and Spawning Bioassay

Contents of soluble protein (SP), vitellogenin (VTG), 17 beta-estradiol (E2), progesterone (Pg), luteinizing hormone (LH), follicle stimulating hormone (FSH) of ovaries anatomized from females subjected to *PcMos* knockdown were determined using a total protein assay kit (Nanjing Jiancheng Bioengineering Institute, Nanjing, China) and VTG/E2/Pg/LH/FSH ELISA Kits (Aviva Systems Biology, Beijing, China) following the specifications of manufacturers. These kits have been previously validated by the manufacturer for cross-reactivity and successfully utilized in *P. canaliculata* [26,44]. Briefly, samples were immersed in 100 mM cold phosphate-buffered saline (PBS, pH = 7.2–7.4) (1:9, *w*/*v*), homogenized using the SKXL homogenizer (Biheng Biotechnology, Shanghai, China), then centrifuged at 12,000× *g* for 30 min at 4 °C (Eppendorf centrifuge 5418R, Hamburg, Germany) to yield supernatants for bioassays. Absorbance at the wavelengths was read in a UV-2550 spectrophotometer (Shimadzu, Tokyo, Japan). Each treatment and control was performed thrice with three independent biological samples.

Female fecundity was examined according to previous methods [1,7]. Wild *P. canaliculata* populations were identified, sampled and reared as described in Section 2.1. Egg masses laid by the same batch of wild-type (wt) females were left to hatch under controlled indoor conditions. The hatchlings were incubated for two months, followed by separate rearing of male and female individuals for another month. Afterwards, adult females were treated with dsPcMos and dsEGFP, respectively, and paired with wt males at a 1:1 ratio. Post 72 h copulation, females were isolated and fed individually, with their total egg production recorded over the next 15 days. All deposited egg clutches were collected, transferred to an open breeding chamber (42 cm × 20 cm × 26 cm, with tap water to maintain humidity) and monitored for an additional 15 days to register hatching rate. Notably, the remaining non-hatched eggs within egg masses were tallied after they were dispersed in 2% sodium hydroxide solution for 1.5 h. Each group consisted of 10 mating pairs, with dsEGFP as the parallel control.

### 2.8. Immunofluorescence and Confocal Imaging

To detect the distribution and localization profiles of PcMos and PcVTG upon dsRNA injection, immunofluorescence was performed according to our previous protocols [1]. The dissected ovaries were fixed in 4% paraformaldehyde (in PBS, *w*/*v*) for 1 h at room temperature, permeabilized with 0.2% TritonX-100 (in PBS, *w*/*v*) for 15 min, and then blocking with 5% bovine serum albumin (BSA) diluted in PBS with 0.1% TritonX-100 for 4 h. After washing in PBS (3 × 5 min), specimens were incubated with rabbit monoclonal anti-PcMos and anti-PcVTG for 24 h at 4 °C, diluted 1000-fold in PBST containing 3% BSA. These primary antibodies were custom-synthesized and purified by GenScript Biotech Corporation (Nanjing, China). After three washes, samples were, respectively, probed using AF488-labeled goat anti-rabbit IgG (H + L) (1:500, Beyotime, Shanghai, China), AF647-labeled goat anti-rabbit IgG (H + L) (1:500, Beyotime, Shanghai, China) at 4 °C for 2 h and counterstained with 1 µg/mL 4′,6-diamidino-2-phenylindole (DAPI, Beyotime, Shanghai, China) for 10 min at room temperature. Post PBS rinsing (3 × 5 min), we mounted specimens on glass slides with antifade medium (Beyotime, Shanghai, China) and signal acquisition was performed on a laser-scanning confocal microscope (LSM 780, Carl Zeiss, Jena, Germany), with images photographed using a Zeiss AxioCam M5Rc digital camera (Zeiss, Jena, Germany). To ensure the reliability of the immunostaining assays, methodological negative controls were routinely performed by substituting the primary antibody with PBS, which resulted in no observable signals, eliminating the possibility of non-specific secondary antibody binding.

### 2.9. Hematoxylin and Eosin (H&E) Staining

Ovary samples were dissected, fixed in 4% paraformaldehyde/0.1 M PBS (pH 7.4) for 24 h at 4 °C, stored in phosphate buffer at 4 °C, dehydrated in an ascending series of ethanol, passed through xylene, and embedded in paraffin. To avoid potential artifacts caused by varying sectioning planes in the highly branched ovarian tissue, multiple sequential sections at varying tissue depths were evaluated and tissue sampling was strictly standardized. Ovarian samples were consistently excised from the mid-region of the main ovarian mass and embedded in a uniform orientation. Furthermore, serial sectioning at 5 μm thickness was performed throughout the embedded tissue blocks (Figure S4) with a microtome (Leica RM2235, Leica Microsystems, Wetzlar, Germany). The serial sections were mounted onto glass slides, dewaxed and stained with hematoxylin-eosin (H&E, G1003, Servicebio, Wuhan, China) as previously described [44]. The slides were observed under a light microscope (Olympus CX31, Olympus, Tokyo, Japan) to examine the histopathological changes.

### 2.10. Homology Modelling and Molecular Docking

The three-dimensional (3D) structures of PcMos and PcVTG were predicted from their respective amino acid sequences using the Swiss Model web server (https://swissmodel.expasy.org/, accessed on 15 May 2026). The dominant conformation with the highest score was considered the definitive structural model for analysis. Structural preprocessing was subsequently manipulated in MGLTools v1.5.6 (https://ccsb.scripps.edu/mgltools/1-5-6/, accessed on 15 May 2026), encompassing water molecule elimination, hydrogen addition, charge assignment, and non-polar hydrogen merging. Afterwards, the refined models were amenable to docking simulations in the ZDOCK program (https://zdock.umassmed.edu/, accessed on 15 May 2026), and each binding model was evaluated using an energy-based scoring function. Among 1000 docking trials, the docking conformation with the lowest binding energy was considered the optimal PcMos-PcVTG complex. Molecular docking and interactions were visualized using PyMOL v.2.5.2 (DeLano Scientific, San Carlos, CA, USA), and the binding free energies (∆Gbind) were calculated using PDBePISA (https://www.ebi.ac.uk/msd-srv/prot_int/cgi-bin/piserver, accessed on 15 May 2026).

### 2.11. Statistical Analysis

All experiments were independently repeated at least three times, and the data were presented as mean ± standard error (SEM). Statistical analyses were performed using SPSS 26.0 (SPSS Inc., Chicago, IL, USA). The Shapiro–Wilk test was used to test the normality of distribution and Levene’s test for checking the homogeneity of variance. If a normal distribution appeared, one-way analysis of variance (ANOVA) followed by Tukey’s post hoc test was utilized to compare the differences among multiple groups. Two-tailed unpaired Student’s *t*-tests were applied to compare the data of two groups. When heteroscedasticity occurred, data were amenable to the Kruskal–Wallis test followed by Dunn’s post hoc test. All graphs were generated using Prism version 9.0.0 (GraphPad Software, San Diego, CA, USA). The statistical significance level (probability value) was set at * *p* < 0.05, ** *p* < 0.01.

## 3. Results

### 3.1. The Bioinformatic Analysis of PcMos

**Figure 1:**
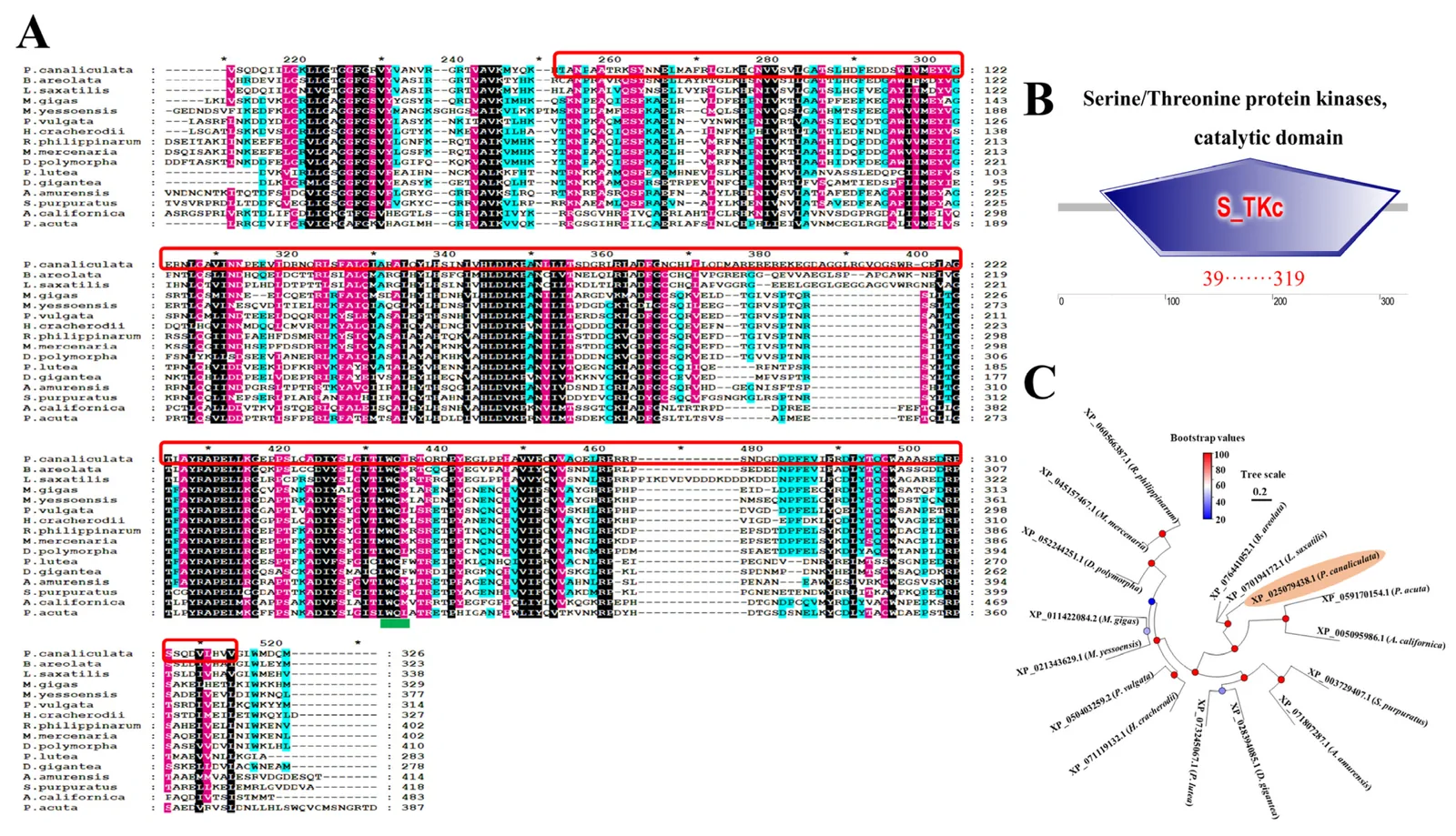
The molecular characterization of PcMos amino acid sequence. (**A**) Multiple sequence alignment of PcMos cognates. Residues shaded in black, pink and cyan are identical (100%), conserved (80%) or similar (60%) in terms of homology. The asterisk (*) denotes an interval distance of 10 aa. Red frames denote the conserved S-TKc domain (39–319 aa) (**B**). The green bar marks a signature motif unique to Mos proteins, WQM/L. The aligned sequence information is retrieved from the NCBI database under accession numbers: XP_025079438.1 [*Pomacea canaliculata*], XP_076441052.1 [*Babylonia areolata*], XP_070194172.1 [*Littorina saxatilis*], XP_050403259.2 [*Patella vulgata*], XP_060566387.1 [*Ruditapes philippinarum*], XP_071119132.1 [*Haliotis cracherodii*], XP_045157467.1 [*Mercenaria mercenaria*], XP_011422084.2 [*Magallana gigas*], XP_052244251.1 [*Dreissena polymorpha*], XP_021343629.1 [*Mizuhopecten yessoensis*], XP_005095986.1 [*Aplysia californica*], XP_073245067.1 [*Porites lutea*], XP_028394085.1 [*Dendronephthya gigantea*], XP_071807287.1 [*Asterias amurensis*], XP_003729407.1 [*Strongylocentrotus purpuratus*], XP_059170154.1 [*Physella acuta*]. (**C**) Phylogenetic relationships between PcMos and its orthologues. Dots at branch nodes represent bootstrap values (1000 replicates). Scale bar indicates the number of amino acid substitutions per site.

Sequence analysis showed the conserved protein kinase domain of PcMos, ranging from 39 to 319 aa (Figure 1B). The S-TKc structural domain and signature motif (WQM/L) were shared among molluscan Mos homologs with high similarity (Figure 1A). The high evolutionary conservation between PcMos and its molluscan counterparts was further illustrated in the dendrogram, where PcMos clustered with the *Babylonia areolata* Mos as one clade and PcMos harbored close phylogeny with orthologues from *B. areolata* (61.96%) and *Littorina saxatilis* (61.47%), with the least identity to that of *Physella acuta* (38.57%) (Figure 1C).

### 3.2. The Spatiotemporal Expression Profiling of PcMos

**Figure 2:**
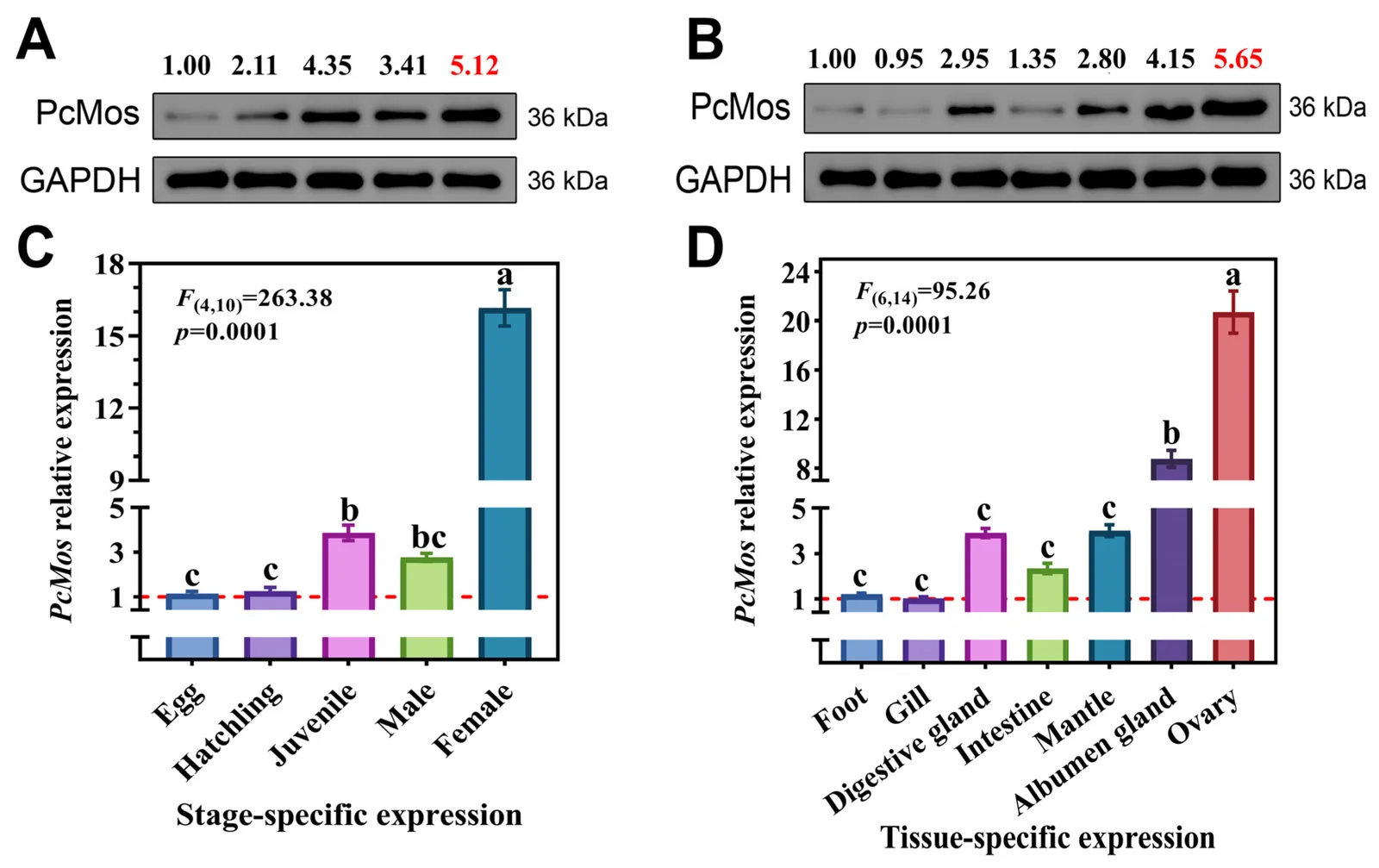
The spatial–temporal expression profiles of PcMos. (**A**,**C**) Relative protein and mRNA levels of PcMos at different development stages. (**B**,**D**) Relative protein and mRNA levels of PcMos in various female tissues. Relative expression levels are normalized to GAPDH with values of egg and foot as calibrators. Each histogram and error bar represents mean ± SEM (*n* = 3). Different lowercase letters denote significant differences among groups (*p* < 0.05, one-way ANOVA followed by post hoc Tukey’s test). Relative protein levels are listed above corresponding bands and calculated as a ratio of grey values, viz., target protein divided by GAPDH.

Despite the constitutive expression across life stages, the predominant abundance of *PcMos* mRNA was observed at the female adult stage, significantly rising by 4.84-fold as compared to that of males (Figure 2C). Notably, at the female stage, *PcMos* was preferentially transcribed in the reproductive tract system, with relative expression peaking in ovarian tissues, followed by the albumen gland tissues (Figure 2D). Similarly, the PcMos protein level was consistent with this female-biased expression trend, being enriched in female adults and their ovarian tissues (Figure 2A,B and Figure S1).

### 3.3. dsPcMos Injection Delivered RNAi Response and Attenuated MAPK Signaling

**Figure 3:**
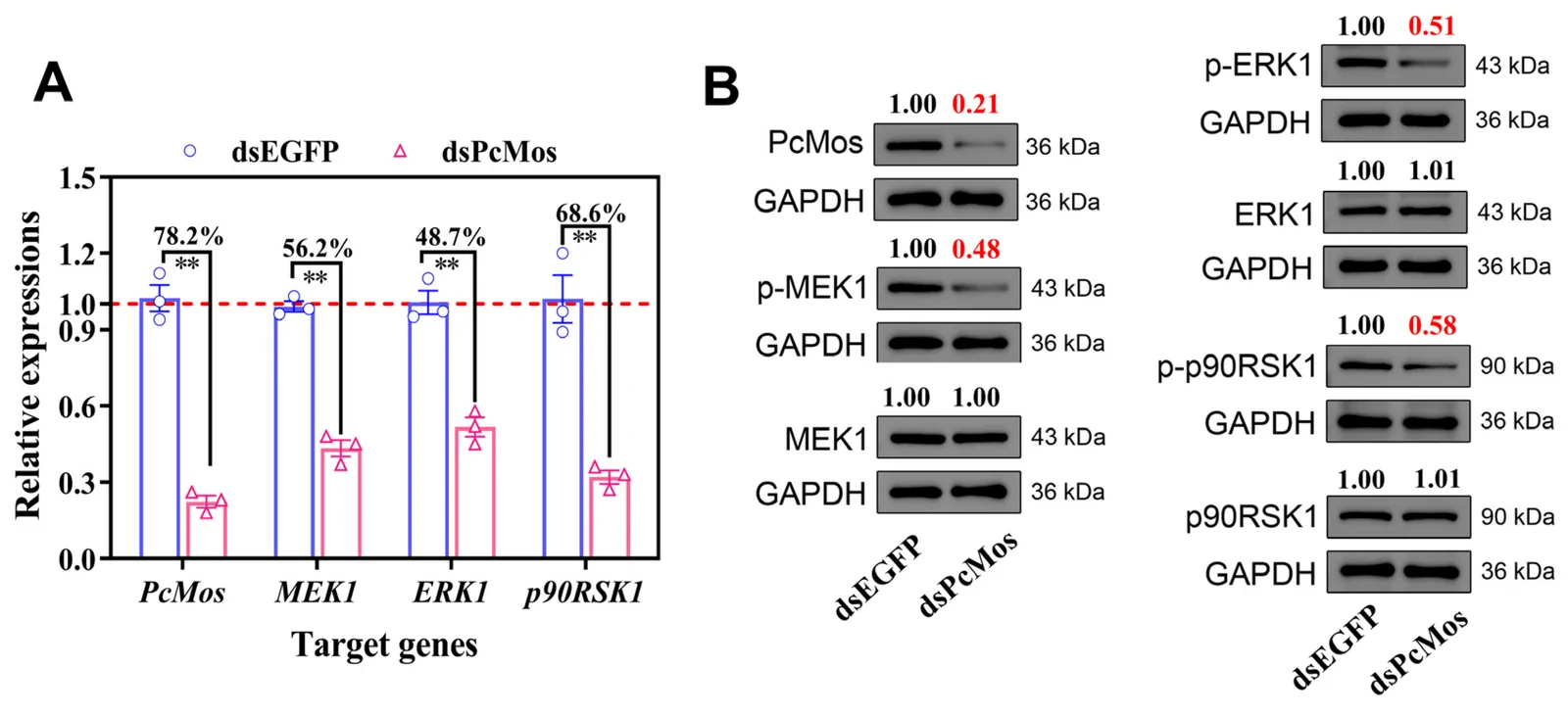
*PcMos* silencing attenuated the MAPK signaling. (**A**) Relative transcript levels of *PcMos*, *MEK1*, *ERK1*, and *p90RSK1* following RNAi treatment. The mRNA levels in the control group (dsEGFP) were used as the reference value and set to 1.0 (indicated by the red dashed line). Data are presented as means ± SEM (*n* = 3), with individual data points displayed as circles (dsEGFP) and triangles (dsPcMos). Statistical comparisons were determined using Student’s *t*-test (** *p* < 0.01). (**B**) Representative blotting images of PcMos, p-MEK1/MEK1 (total), p-ERK1/ERK1 (total), and p-p90RSK1/p90RSK1 (total). Relative grey values are normalized to GAPDH and marked above the corresponding bands. Protein expression levels of the dsEGFP group were set to 1.00 as the calibrator.

Females receiving dsPcMos administration displayed a robust RNAi response, as revealed by the pronounced suppression of PcMos expression at both the mRNA (78.2% ↓, *t* = 13.95, df = 4, *p* < 0.01) and protein levels (79.0%↓) versus the dsEGFP treatment (Figure 3A,B). The qRT-PCR analysis showed that MAPK signaling was strongly dampened by the dsPcMos treatment (Figure 3A), with a significant decrease in transcript levels for MEK1 (56.2% ↓ *t* = 14.32, df = 4, *p* < 0.01), ERK1 (48.7% ↓ *t* = 8.14, df = 4, *p* < 0.01) and p90RSK1 (68.6% ↓ *t* = 7.25, df = 4, *p* < 0.01). In addition, Western blotting revealed that *PcMos* knockdown did not alter the total protein abundance of MEK1/ERK1/p90RSK1. However, their phosphorylation levels markedly dropped by 52.0%, 49.0% and 42.0%, respectively, indicating that the MAPK signaling was attenuated at the phosphorylation level (Figure 3B and Figure S2).

### 3.4. dsPcMos Treatments Perturbed Endocrine Hormone Homeostasis

**Figure 4:**
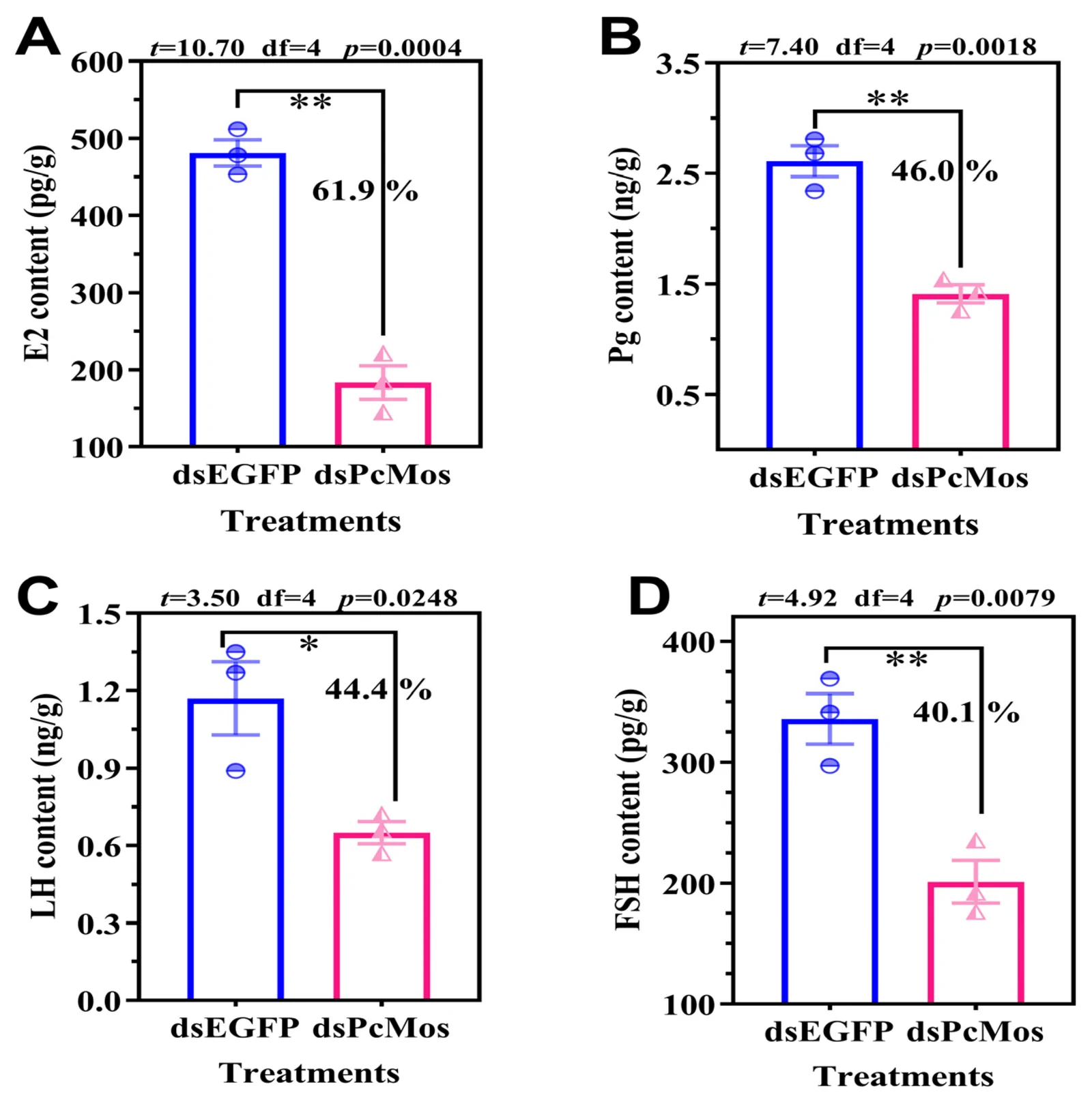
Alterations in contents of female endocrine hormones post dsPcMos treatments. (**A**) E2 (pg/g protein); (**B**) Pg (ng/g protein); (**C**) LH (ng/g protein); (**D**) FSH (ng/g protein). Each histogram and error bar represents the mean ± SEM (*n* = 3). Statistical comparisons are performed using Student’s *t*-test, and asterisks denote significant differences (* *p* < 0.05, ** *p* < 0.01).

The contents of ovarian hormones, including E2, Pg, LH and FSH all markedly declined upon *PcMos* silencing, down by 61.9%, 46.0%, 44.4% and 40.1%, respectively, relative to the control group (Figure 4).

### 3.5. Effects of PcMos Knockdown on Yolk Accumulation

**Figure 5:**
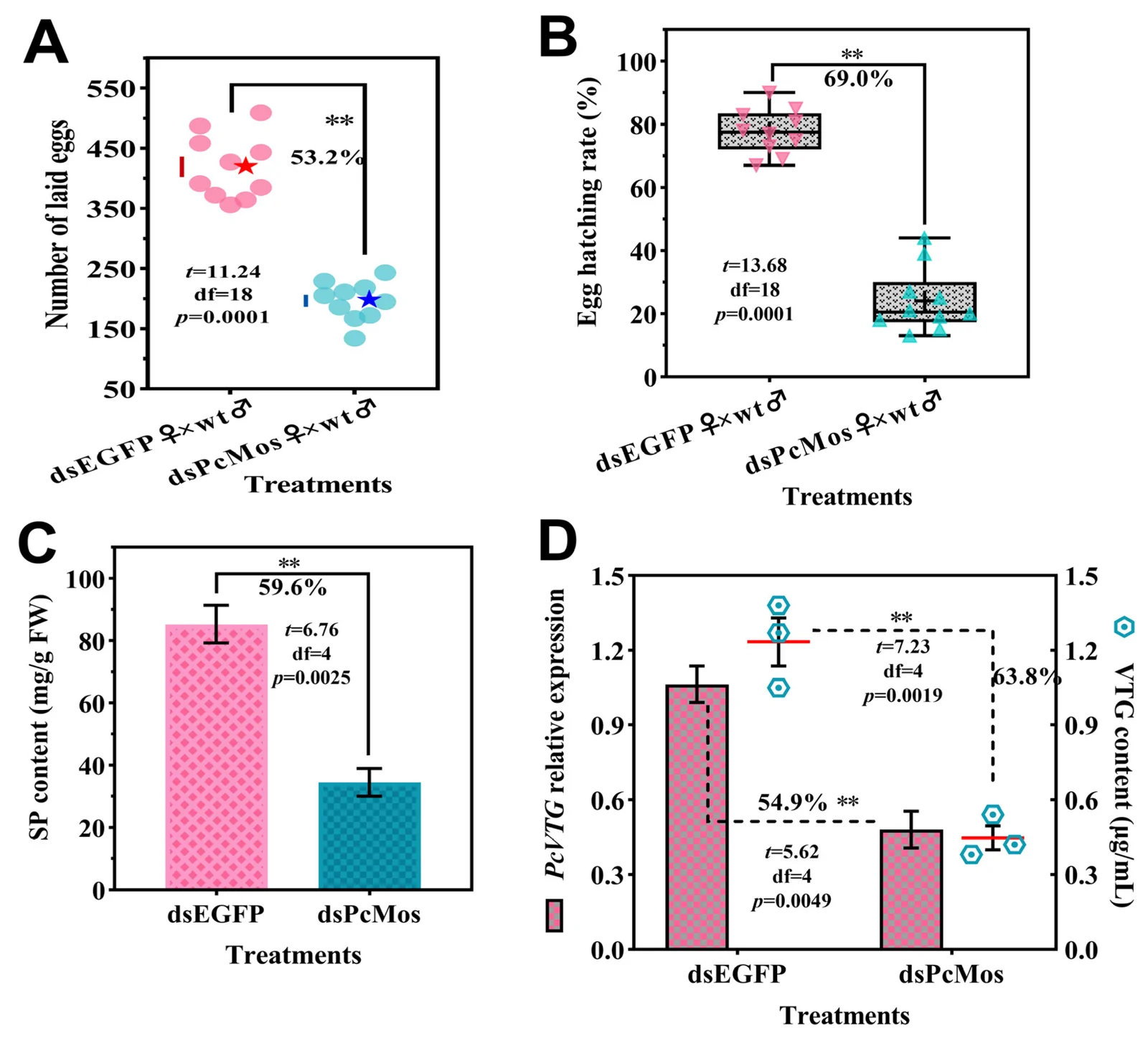
Effects of PcMos knockdown on egg-laying (*n* = 10) (**A**), egg hatchability (*n* = 10) (**B**), SP content (mg/g fresh weight) (*n* = 3) (**C**), *PcVTG* transcript level and content (μg/mL protein) (*n* = 3) (**D**). Each pink/cyan circle, triangle and cyan hexagon denotes a raw data. Red/blue vertical lines and stars mark error bars and means, respectively. Black horizontal lines represent medians, with “+” as means. Boundaries of the box plot indicate 25/75th percentiles, and whiskers indicate maximum and minimum. Red horizontal lines represent means. Each histogram and error bar denotes the mean ± SEM. Statistical comparisons are performed using Student’s *t*-test (** *p* < 0.01).

**Figure 6:**
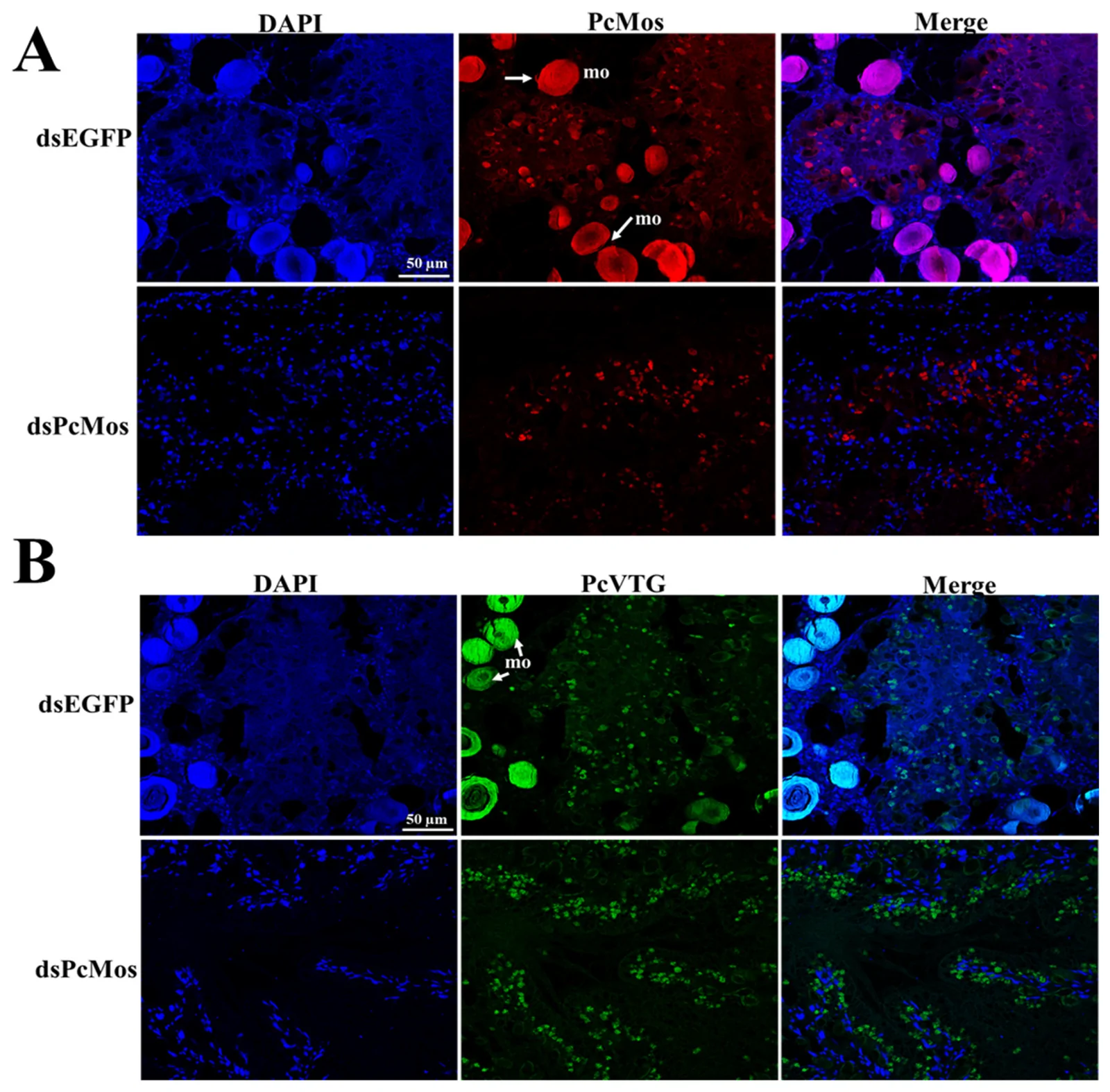
Representative immunofluorescence images of PcMos protein (**A**) and PcVTG protein (**B**) in the ovarian tubule upon dsRNA injections. The localization and expression are illustrated by fluorescent intensity. The oocyte nuclei, PcMos antibody and PcVTG antibody are labelled by DAPI (blue), Alexa-647 (red) and Alexa-488 (green), respectively. White arrows represent mature oocytes (mo). Scale bar, 50 µm.

dsPcMos injections significantly reduced ovarian contents of soluble protein, VTG as well as the VTG transcript level as compared to the dsEGFP group (Figure 5C,D), with significant diminutions of 59.6%, 63.8% and 54.9%, respectively. In ovarian tubules, virtually no mature oocytes alongside sparse distribution and weak accumulation of PcMos were noticed in *PcMos*-deficient females, while the dsEGFP group harbored an even and dense immunofluorescence signal for PcMos in ovarian tubules and mature oocytes (Figure 6A). Similarly, nil mature oocytes plus sporadic distribution and faint signal intensity of VTG were detected post dsPcMos treatment, differing from the abundant and intense immunoreactivity in the dsEGFP-treated ovaries packed with developing and mature oocytes (Figure 6B). The molecular docking analysis revealed strong interactions and high binding affinity between VTG and PcMos, primarily mediated by hydrogen bonds and hydrophobic contacts, with a binding free energy (ΔGbind) of -13.7 Kcal/mol (Figure S3).

### 3.6. PcMos Silencing Compromised Ovarian Development and Female Fecundity

**Figure 7:**
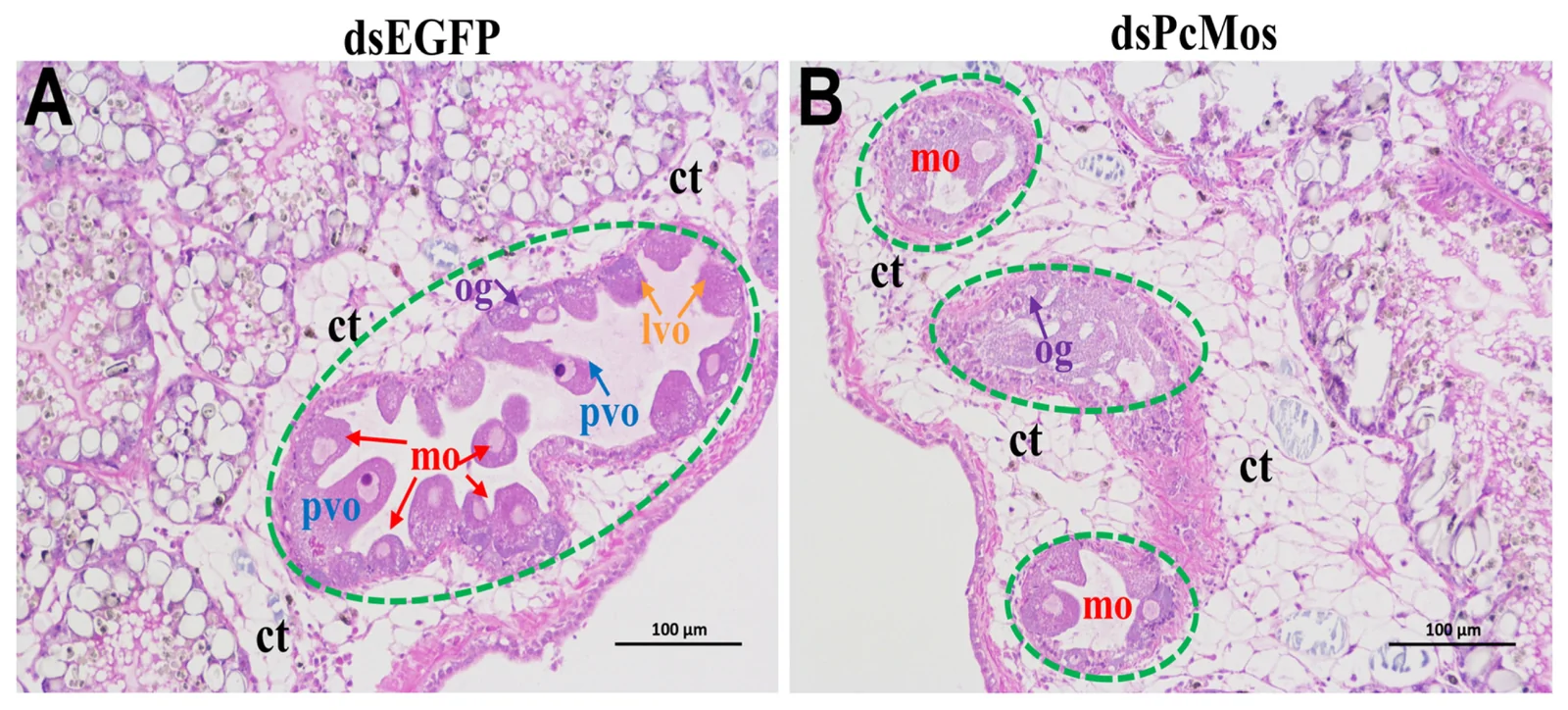
H&E staining of ovarian tissues of *P. canaliculata* subjected to dsEGFP treatment (**A**) and *PcMos*-RNAi (**B**). Ovarian tubules are circled by the green dash line. ct, connective tissue; mo, mature oocyte; pvo, previtellogenic oocyte; lvo, late vitellogenic oocyte; og, oogonium. Scale bar, 100 μm.

As shown in Figure 5 and Figure 7, the female reproductive fitness was impaired by *PcMos* silencing in three aspects. First, females subjected to the dsPcMos treatment significantly diminished the oviposition of copulatory pair, with a 53.2% decrease in the number of eggs laid compared to the control mating group (Figure 5A). Second, the egg hatching rate was significantly reduced upon *PcMos* depletion, down by 69.0% relative to the control (Figure 5B). Third, the arrested oocyte growth, negligible mature oocytes and distinct compartmentalization were visualized in dsPcMos-treated ovaries versus the well-developed and intact phenotype of the control group, as presented in the H&E staining (Figure 7). Furthermore, analysis of serial sections at varying tissue depths (Figure S4) demonstrated that the atrophic and fragmented tubules in the dsPcMos group did not converge into continuous structures in deeper planes. The overall structural degradation and severe depletion of developing oocytes were consistently observed across all serial sections in the dsPcMos group compared to the oocyte-rich contiguous tubules in the dsEGFP group.

## 4. Discussion

**Figure 8:**
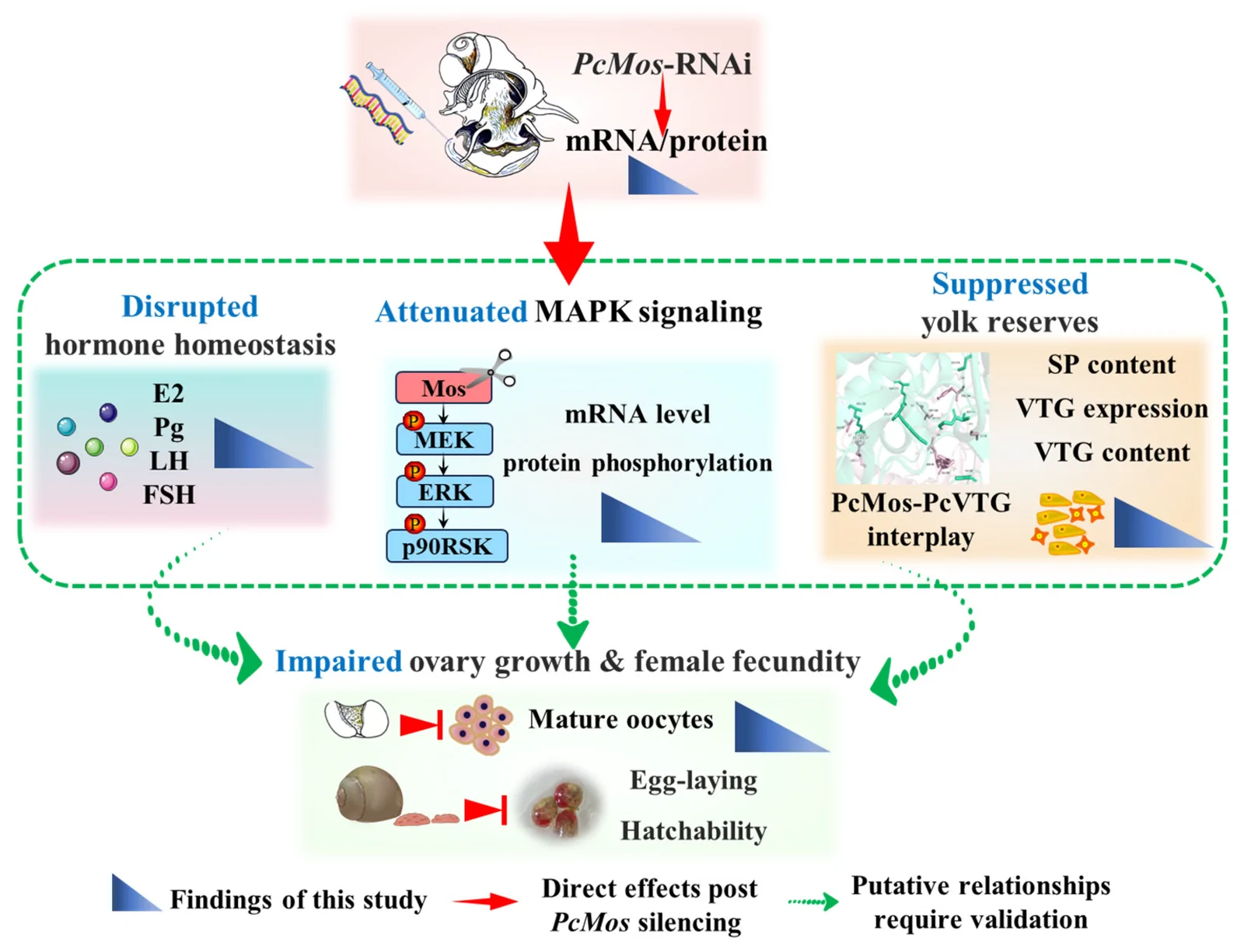
Schematic illustration of the proposed mechanisms linking *PcMos* to female fecundity in *P. canaliculata*. Blue triangles denote the specific phenotypes and molecular downregulations demonstrated in this study. Solid red arrows signify the direct downstream effects observed post *PcMos*-RNAi (i.e., attenuation of MAPK signaling, disruption of endocrine hormones, and suppression of yolk reserves). Green dashed arrows represent hypothesized associations and potential regulatory pathways leading to impaired ovary growth and reduced fecundity. These dashed lines imply putative relationships that warrant further mechanistic validation.

Gametogenesis and reproduction are fundamental biological processes that sustain population persistence across dioecious organisms [1,45]. The high prolificacy of *P. canaliculata* fuels its rapid invasiveness and establishment, raising public concerns and highlighting the need for precision pest management (PPM) strategies like RNAi [46]. Mos is a crucial regulator of oocyte meiotic progression in female animals [36,37] and thus holds considerable promise as the target of RNAi-mediated pest control. In this study, we assumed that *PcMos* exerted reproduction-related functions in *P. canaliculata* and leveraged RNAi to corroborate its involvement. As expected, the dsPcMos injection induced a robust RNAi response and *PcMos* knockdown was associated with alterations in female vitellogenesis, oogenesis, and fecundity, concurrent with changes in MAPK signaling, vitellogenic and hormonal profiles (Figure 8). However, further studies are required to establish the direct causal mechanisms linking these observations.

Mos proteins are phylogenetically conserved across the animal kingdom, as reported in the insect, mammal, cnidarian and molluscan phyla [36,37,47,48]. Resembling the sequence properties of pearl mussel C-Mos [36] and sawfly Mos [48], PcMos also featured the S-TKC structural domain and a unique motif. Moreover, the MAPK kinase activity of Mos [49] plus the reproductive function of the S-TKC domain [36] imply that Mos is involved in cellular phosphorylation and reproductive processes. Spatiotemporally, PcMos was favorably enriched in individual females and female gonads but deficient in eggs, hatchlings, males and somatic tissues. This tendency is also present in *Clytia* and triangle sail mussel (*Hyriopsis cumingii*), where the expression of Mos is restricted to female gonads [36,47]. In humans, Zeng et al. [50] also found low transcript level of Mos in embryos and somatic tissues. Conversely, Mos mRNA is highly transcribed in human oocytes, with its protein actively translated during oocyte maturation [51]. Similarly in the mollusc *H. cumingii*, in situ hybridization signals of *C-Mos* are detected in oocytes, oocyte nuclei and oogonia [36]. In line with this pattern, we observed copious and intense immunoreactivity of PcMos in growing/ mature oocytes and ovarian tubules, which was supported by findings from *Clytia* [47] and sawfly [48] oocytes. Overall, PcMos displayed the oocyte-specific and female-biased expression pattern, indicative of its contribution to oocyte maturation, ovarian development and female fecundity. In addition to the expected concurrent expression trends of PcMos mRNA and protein, we also detected their discrepant fold changes, which may be attributed to posttranscriptional regulation, posttranslational modifications and differential degradation rates of mRNA and protein [1,3].

A growing body of literature has evidenced that Mos operates as a MEK kinase upstream of the MAPK pathway [47,48,49] and Mos-MAPK signaling executes conserved and pleiotropic roles during oocyte meiosis in mouse [37,49], frog [35], sawfly [48] and cnidarian [47]. This work echoed the fact that the MAPK pathway functioned downstream of *PcMos*, with transcript levels of *MEK1/ERK1/p90RSK1* all significantly reduced upon *PcMos* suppression. Concurrent with this tendency, the phosphorylation levels of MEK1/ERK1/p90RSK1 substantially declined. Based on the unaltered total protein abundance, decreased phosphorylated protein and mRNA levels of MAPK members, it was tempting to speculate that *PcMos* activated the MAPK pathway at both post-translational (i.e., via phosphorylation) and transcript levels but not at the translation level. Intriguingly, this regulatory pattern resembled the reports of Lawan et al. [52] and Liu et al. [53], where MAPK is also regulated transcriptionally and post-translationally by other molecular elements. The MAPK cascade partakes in the reproductive development of molluscan species [33]. In this study, we speculated that the reductions in VTG deposition, mature oocytes and egg hatchability can be ascribed to the attenuated MAPK phosphorylated activity in that MAPK signaling activation is requisite for oocyte cell cycle, oocyte maturation and embryo development in mammals [30,51]. This argument was reinforced by the findings that mutant Mos proteins failed to activate the ERK1/2 cascade and decreased the phosphorylation level of ERK1/2 in mouse oocytes [51]. Likewise, knockout of *Mos* or *Erk1/2* in mice results in abnormal oocyte meiosis and early embryo developmental arrest [54,55,56]. The RNAi against *H. cumingii C-Mos* also resulted in the downregulation of both *ERK* and *P90rsk*, signifying that *C-Mos* is involved in primordial germ cell proliferation and oocyte cell cycle through regulating the MAPK pathway [36].

An early study has pointed out that perturbing key reproductive hormones directly affects snail reproduction [26]. Herein, the contents of E2/Pg/LH/FSH underwent significant decreases post *PcMos* knockdown. The synchronized decline of all major hormones in females reflected that the overall endocrine hormone homeostasis was perturbed by *PcMos*-RNAi, which may be associated with the reproductive loss. Furthermore, we noticed that E2 displayed the largest reduction, suggesting that E2 was the hormone most severely impacted by *PcMos* silencing. In non-mammalian species, E2 acts as a decisive inducer of ovarian growth, and promotes vitellogenin synthesis during ovarian development and oogenesis [57]. Maintenance of sufficient E2 correlates with normal ovary functions. The intense fluctuation of E2 content can provoke various endocrine disorders [26,44,58], which manifested as the defective ovarian phenotype, arrested oocyte growth and impeded VTG accumulation in this study. In molluscs, mounting evidence confirms that oocyte growth through vitellogenesis is affected by the endocrine hormone balance [59,60,61]. Our findings corroborate this view. Aligned with our results, Xue et al. [26] reported that exposure of *P. canaliculata* to tamoxifen caused a dramatic shortage of E2/Pg/LH/FSH and endocrine hormone imbalance, which reduced the number and quality of fertilized eggs. In this sense, dsPcMos presumably performed analogously to an endocrine disruptor that adversely impacts the endocrine system and triggers hormonal dysfunction.

Aside from being mandatory structural and functional building blocks of tissues, proteins serve as necessary macronutrients for life activities of living organisms [62]. In oviparous animals, vitellin (Vn) is the main component of the egg yolk [63]. Well-documented as a female-specific lipoglycophosphoprotein and indicator of reproductive fitness, vitellogenin (VTG) constitutes the precursor of Vn and provides essential nutrition for oocyte maturation and fertilized eggs [26,64,65]. In this study, ovarian soluble protein (SP) and VTG levels as well as *PcVTG* mRNA substantially dropped in dsPcMos-treated females, where weak and sparse immunoreactivity of VTG was also noticeable. These adverse outcomes reflected that *PcMos* depletion resulted in deficient VTG accumulation and yolk reserves of the ovary. Hence, the reduced nutrient deposition may restrict the resources shifted to oocyte and embryonic development, which can account for the impaired ovarian phenotype and arrested oocyte maturation, concomitant with suppressed egg-laying and hatchability.

In oviparous females, the gametes (eggs or ova) are produced in the ovary via a process designated as oogenesis [66]. Vitellogenesis represents one of the most vital processes during which proteins, carbohydrates and lipids indispensable for embryonic development are synthesized and stored inside the developing oocytes [64,67]. Oogenesis and vitellogenesis are tightly and precisely regulated through a plethora of hormonal signals, nutrient sensors and signaling cascades [68,69]. Our findings lent credence to this notion, given that (1) phosphorylation activation of MAPK signaling was attenuated by *PcMos* silencing; (2) endocrine homeostasis was perturbed by diminished E2/Pg/LH/FSH levels upon *PcMos* knockdown; (3) *PcMos*-RNAi suppressed yolk reserves via reducing the deposition of VTG and SP. The three modulatory axes operate collaboratively to affect female reproductive fitness and their disruption may impede vitellogenesis and oogenesis, resulting in impaired ovary development, egg-laying alongside hatchability (Figure 8). These alterations of reproductive aspects are aligned with the reports from mammals that *Mos*-deleted females exhibit low fertility, abnormal germ cell division and deformed ovaries, accompanied by fertilized eggs unable to develop into a viable embryo [37,54,55]. In addition, the crosstalk among the three regulatory pathways and interplay of reproductive proxies should be clarified. This intricate interdependence is demonstrated in molluscs. Injection of E2 (1–1000 nmol/animal) can induce VTG within 72 h in *Mya arenaria* and *Elliptio complanata*, suggesting that vitellogenesis is an indicator of endocrine effects and controlled by E2 [59,70]. However, in *P. canaliculata*, it remains unclear whether and how the MAPK signaling modulates VTG accumulation or endocrine hormone synthesis, and whether and how endocrine hormones are implicated in vitellogenesis. These questions warrant further exploration.

Conventional control of *P. canaliculata* primarily resorts to chemical agents albeit at the cost of nontarget ecotoxicity to fish [12,13]. By curbing key genes pertaining to growth, development and metabolism, the RNAi technique has been embraced in precision pest management (PPM) and has been proven an effective alternative for invasive pest management [46,71]. This study extends our prior work [1], where *Pcbol* is characterized as a central player in male spermatogenesis and fertility. Since *P. canaliculata* is a dioecious animal with high prolificacy and both bol and Mos genes are pivotal for gamete meiotic progression in most animals, our findings identify the two molecular keys that modulate gametogenesis and govern reproductive physiology of *P. canaliculata* from a bisexual perspective. Compared to the single gene RNAi, targeting the two genes more effectively suppresses reproduction in both sexes. More importantly, this study also manifested strong in silico binding affinity between PcMos and PcVTG, and both proteins exhibited concurrent distribution in vitellogenic oocytes. Beyond downregulating PcVTG expression, it was likely that *PcMos*-RNAi may directly disrupt PcMos–PcVTG interactions. Considering VTG as a well-established molecular marker of vitellogenesis [60] and the reproductive importance of PcMos and Pcbol, formulations of the dsRNA cocktail by co-silencing *PcVTG* + *PcMos*, *Pcbol* + *PcMos* or *Pcbol* + *PcVTG* + *PcMos* can exert pleiotropic detrimental effects on vitellogenesis, oogenesis and spermatogenesis, thereby synergistically exacerbating reproductive failure. Collectively, our studies pave the way for the viable application of a genetics-based sterile technique and PPM strategy in *P. canaliculata* management.

## 5. Conclusions

In summary, PcMos was phylogenetically conserved among molluscan homologs and presented the female/ovary-biased expression pattern. *PcMos*-RNAi significantly reduced levels of E2, Pg, LH, FSH, SP and VTG in ovaries relative to the dsEGFP control. In addition, VTG expression and MEK1/ERK1/p90RSK1 phosphorylation were all consistently downregulated post *PcMos* silencing. These lines of evidence revealed that dsPcMos-elicited fecundity loss could be attributed to compromised endocrine hormone homeostasis and yolk accumulation alongside the dampened MAPK pathway. *PcMos* not only proved to be the molecular switch for female fecundity but also functioned as a hub gene interlinked endocrine regulation, vitellogenic process and cellular signaling with reproductive performance. To further evaluate the application efficacy and stability of the dsPcMos-based sterility agent in the field, ongoing studies will encapsulate the naked dsPcMos with protective nanomaterials or formulate it with an attractant bait to enable the spraying or immersion delivery.

## Data Availability

Data are contained within the article or Supplementary File.

## Supplementary Materials

The following supporting information can be downloaded at: https://www.mdpi.com/article/10.3390/biology15191774/s1, Figure S1: The uncropped original Western blotting images for Figure 2A (left) and Figure 2B (right). These assays were conducted on 2026/07/09; Figure S2: The uncropped original Western blotting images for Figure 3B. These assays were conducted on 2026/07/07; Figure S3: In-silico docking model based on protein structures of PcVTG (pink) and PcMos (green). The peptide binding interface was circled and enlarged in the right panel, where hydrogen bonds (yellow) and salt bridges (red) were labelled by dashed lines. Distances (Å) of residue interactions were presented numerically. The binding energy was calculated as ΔGbind= −13.7 Kcal/mol; Figure S4. Serial histological sections of the ovarian tissues in *P. canaliculata* following dsEGFP (A, B, C) and dsPcMos (D, E, F) treatments. The sequential images represent increasing tissue depth. In the dsEGFP group (left panels), large, continuous, and thick-walled ovarian tubules densely packed with developing oocytes are consistently maintained across all depth planes. In the dsPcMos group (right panels), the shrunken, disjointed tubules surrounded by extensive connective tissue persist across all serial sections. Notably, these separated tubules do not connect into continuous structures in deeper planes. The consistent depletion of oocytes and thinning of the germinal epithelium across all depths confirm that the observed ovarian atrophy is a true biological effect of PcMos knockdown rather than a sectioning plane artifact. Scale bars, 100 μm; Table S1: The gene-specific primers for qRT-PCR assays and dsRNA synthesis; Table S2: Primary antibody information for Western blotting; Text S1: Quality control for ovarian tissue purity. See refs. [72,73].

## Abbreviations

Moloney sarcoma oncogene (Mos), hematoxylin and eosin staining (H&E staining), quantitative real-time polymerase chain reaction (qRT-PCR), enzyme-linked immunosorbent assay (ELISA), mitogen-activated protein kinase (MAPK), vitellogenin (VTG), 17 beta-estradiol (E2), progesterone (Pg), luteinizing hormone (LH), follicle stimulating hormone (FSH), phosphate-buffered saline (PBS), bovine serum albumin (BSA), 4′,6-diamidino-2-phenylindole (DAPI), RNA interference (RNAi), soluble protein (SP), amino acid (aa), base pair (bp), probability value (p), enhanced green fluorescence protein (EGFP), double-stranded RNA (dsRNA), sodium dodecyl sulfate polyacrylamide gel electrophoresis (SDS-PAGE), PBST (phosphate-buffered saline with 0.1% Tween 20), precision pest management (PPM), glyceraldehyde-3-phosphate dehydrogenase (GAPDH), wild type (wt).
