# Supplementary material for: Molecular Characterization and Functional Analysis of Pomacea canaliculata Mos Gene (PcMos): A Molecular Switch of Female Fecundity
Source: Biology (Basel). 2026 Oct 6;15(19):1774. doi: 10.3390/biology15191774 (PMC13664547; doi:10.3390/biology15191774)
Supplement: Supplementary file 1 [file biology-15-01774-s001.zip › biology-4550752-supplementary.pdf]

Supplementary Materials

Table S1. The gene-specific primers for qRT-PCR assays and dsRNA synthesis.

Gene name	GenBank Accession No.	Sequences (5′–3′)	Application	Size (bp)
serine/threonine-protein kinase mos-like (*PcMos*)	XM_025223653.1	Forward: taatacgactcactatagggTTTTACACGTGGTGGGGCTG Reverse: taatacgactcactatagggCGTGTAACTACGTGGAAACA	dsRNA	354
enhanced green fluorescent protein gene (*EGFP*)	MN443913.1	Forward: taatacgactcactatagggGCGAGGGCGATGCCACCTAC Reverse: taatacgactcactatagggCACGCTGCCGTCCTCGATGT	dsRNA	431
serine/threonine-protein kinase mos-like (*PcMos*)	XM_025223653.1	Forward: GATGGCGTTCAGACTAGGAC Reverse: ACGTACTCCATGACGATCCA	qRT-PCR	105
glyceraldehyde-3-phosphate dehydrogenase-like (*GAPDH*)	XM_025226527.1	Forward: CAACCTCAAAACCGATGCCA Reverse: GACAAAGCGATTAGTCAGTGGA	qRT-PCR	184

mitogen-activated protein kinase kinase 1-like (MEK1)	XM_025253255.1	Forward: TACATCTCCCATCATCCCCC Reverse: GCCCTAGACACAGAGCATTC	qRT-PCR	93
mitogen-activated protein kinase 1-like (ERK1)	XM_025233290.1	Forward: TCGGCCCAAGATACACAAAC Reverse: ATCTCTCGTAACGTTCGCTG	qRT-PCR	154
ribosomal protein S6 kinase beta-1-like (p90RSK1)	XM_025237113.1	Forward: GCATGCACCATTGGAAAGAC Reverse: CGGGAGAAGAATGGACTTGG	qRT-PCR	114

Note: Underlined lowercase letters indicated T7 promoter sequences.

Table S2. Primary antibody information for western blotting.

Protein names	Catalogue number & manufacture	Phosphorylation sites	Dilutions
GAPDH Rabbit Monoclonal Antibody	AF1186, Beyotime, Shanghai, China	—	1:2000
MEK1 Rabbit Monoclonal Antibody	AG2956, Beyotime, Shanghai, China	—	1:500
Phospho-MEK1 Rabbit Monoclonal Antibody	AF2578, Beyotime, Shanghai, China	Thr292	1:1000
ERK1 Rabbit Polyclonal Antibody	AF0144, Beyotime, Shanghai, China	—	1:1000
Phospho-ERK1 Rabbit Monoclonal Antibody	AF1891, Beyotime, Shanghai, China	Thr202/Tyr204	1:1000
p90RSK1 Rabbit Polyclonal Antibody	AR861, Beyotime, Shanghai, China	—	1:500
Phospho-p90RSK1 Rabbit Monoclonal Antibody	AR865, Beyotime, Shanghai, China	Ser380	1:1000
PcMos Rabbit Monoclonal Antibody	synthesis compound, GenScript Biotech Corporation, Nanjing, China	—	1:500

Text S1 Quality control for ovarian tissue purity

To rigorously ensure the purity of the ovarian samples and rule out digestive gland contamination, a specific molecular quality control step was implemented prior to downstream analyses. It is well-documented that specific symbiotic corpuscles reside exclusively within the epithelial cells of the digestive gland in this species [72,73]. Therefore, we utilized the 16S rRNA gene of this symbiont (Cyanobacterium sp. MBIC10216, GenBank accession: B058249.1) as a negative molecular marker for ovarian purity.

We performed qRT-PCR on the dissected ovarian samples using specific primers designed for this 16S rRNA target (Forward primer: 5’-ATACTAGGCGTAGTGCTGTAAAA -3’; Reverse primer: 5’-AGGATGTCAAGCCTTGGTAAG -3’; product length: 177 bp). The quality control criteria were strictly defined: if high expression of the 16S rRNA was detected in a given sample, it indicated potential digestive gland cross-contamination, rendering the

corresponding hormone and gene expression results unreliable; such contaminated samples were discarded and the sampling procedure was repeated. Conversely, the absence or negligible expression of this 16S rRNA confirmed that the sample was successfully isolated and free of digestive gland tissue. All ovarian samples utilized for the final data analysis in this study showed negligible 16S rRNA expression, thereby rigorously validating their purity and the reliability of our molecular results.

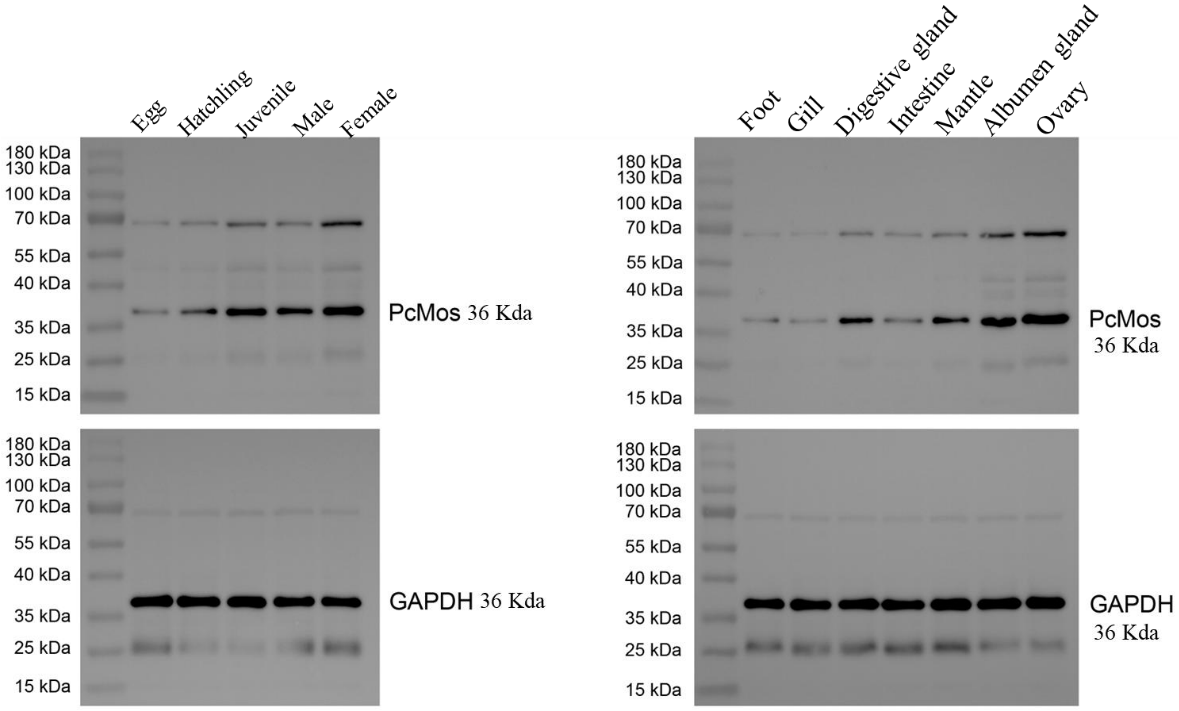

Figure S1. The uncropped original western blotting images for Figure 2A (left) and Figure 2B (right). These assays were conducted on 2026/07/09.

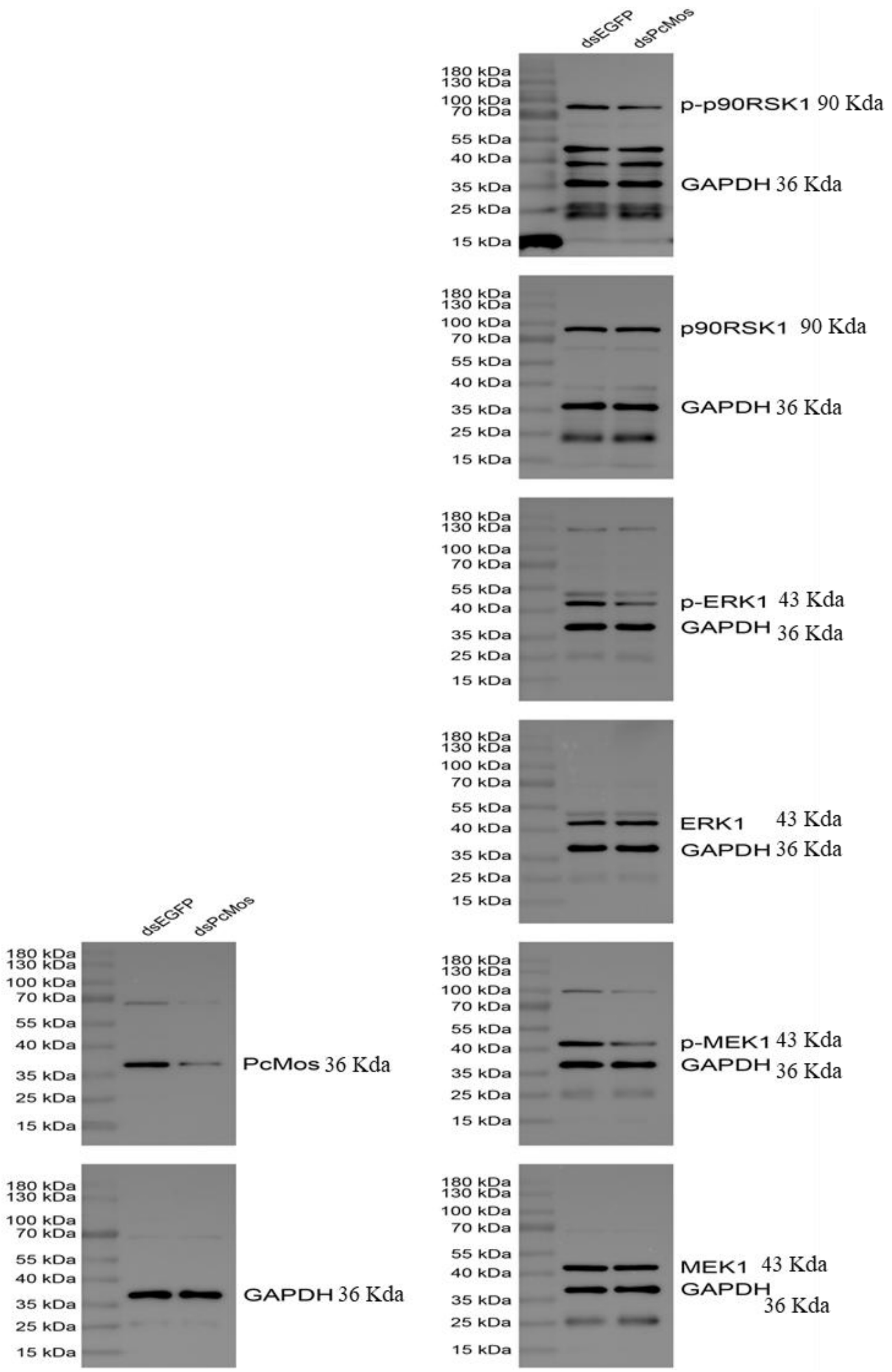

Figure S2. The uncropped original western blotting images for Figure 3B. These assays were conducted on 2026/07/07.

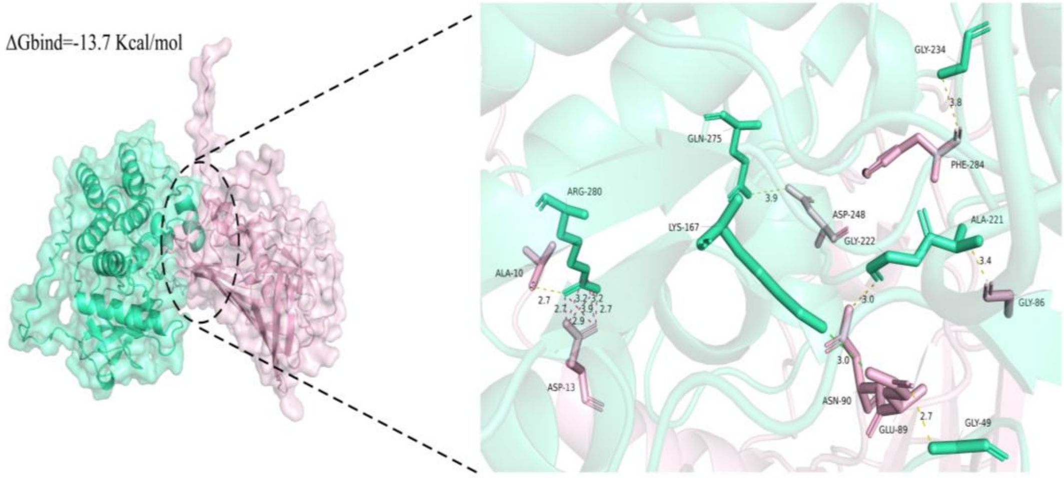

Figure S3. In-silico docking model based on protein structures of PcVTG (pink) and PcMos (green). The peptide binding interface was circled and enlarged in the right panel, where hydrogen bonds (yellow) and salt bridges (red) were labelled by dashed lines. Distances (Å) of residue interactions were presented numerically. The binding energy was calculated as ΔGbind= −13.7 Kcal/mol.

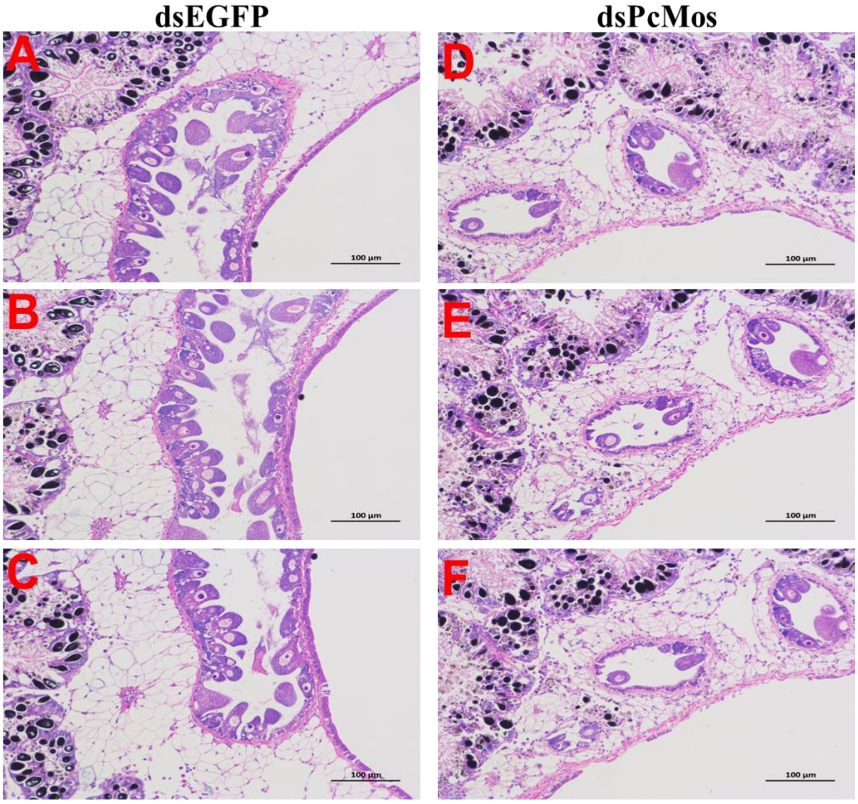

Figure S4. Serial histological sections of the ovarian tissues in *P. canaliculata* following

dsEGFP (A, B, C) and dsPcMos (D, E, F) treatments. The sequential images represent increasing tissue depth. In the dsEGFP group (left panels), large, continuous, and thick-walled ovarian tubules densely packed with developing oocytes are consistently maintained across all depth planes. In the dsPcMos group (right panels), the shrunken, disjointed tubules surrounded by extensive connective tissue persist across all serial sections. Notably, these separated tubules do not connect into continuous structures in deeper planes. The consistent depletion of oocytes and thinning of the germinal epithelium across all depths confirm that the observed ovarian atrophy is a true biological effect of PcMos knockdown rather than a sectioning plane artifact. Scale bars, 100 μm.
